# Age and Gender Differences in Psychological Distress among African Americans and Whites: Findings from the 2016 National Health Interview Survey

**DOI:** 10.3390/healthcare6010006

**Published:** 2018-01-17

**Authors:** Daphne C. Watkins, Natasha C. Johnson

**Affiliations:** 1 School of Social Work, University of Michigan, Ann Arbor, MI 48109, USA; 2 School of Social Work and Department of Psychology, University of Michigan, Ann Arbor, MI 48109, USA; ncjohns@umich.edu

**Keywords:** African Americans, age, gender, race, psychological distress, Whites

## Abstract

Previous studies report a race and mental health paradox: Whites score higher on measures of major depression compared to African Americans, but the opposite is true for psychological distress (i.e., African Americans score higher on distress measures compared to Whites). Independently, race, age, and gender outcomes for psychological distress are well documented in the literature. However, there is relatively little research on how psychological distress interferes with the lives of African Americans and Whites at the intersection of their various race, age, and gender identities. This study uses data from the 2016 National Health Interview Survey to examine age and gender differences in psychological distress and how much psychological distress interferes with the lives of African Americans and Whites. Our study findings are contrary to the paradox such that young White women (M = 3.36, SD = 1.14) and middle-aged White men (M = 2.55, SD = 3.97) experienced higher psychological distress than all other race, age, and gender groups. Psychological distress interference was relatively high among the high distress groups, except for older African American men (M = 1.73, SD = 1.05) and young African American women (M = 1.93, SD = 0.95). Implications for studies that consider cultural experiences of psychological distress, and how it impacts different demographic groups are discussed.

## 1. Introduction

Large epidemiologic studies accentuate variations in understanding the experiences of depression among African Americans compared to Whites. Specifically, there are concerns about whether mental health questionnaires capture the most accurate assessment of depression among marginalized groups, compared to non-marginalized groups. These concerns exist for a range of mental health questionnaires, such as the highly structured, lay interviewer-administered World Health Organization Composite International Diagnostic Interview (WHO-CIDI) and the semi-structured, clinician-administered World Mental Health Structured Clinical Interview for the Diagnostic and Statistical Manual of Mental Disorders ((DSM), WMH SCID 2000), which are both tied to DSM criteria. For example, previous studies have examined issues such as interviewer and gender bias in making clinical decisions about African Americans [1]; the operationalization of DSM depression criteria in the CIDI and SCID [2,3]; mental health stigma [4,5]; and mental health literacy among African Americans [6,7]. In the past, these issues have made it difficult for African Americans to report depression symptoms, especially to interviewers and clinicians who are not African American. 

Though previous studies confirm that rates of major depressive disorder (MDD) are lower for African Americans compared to Whites, some researchers have argued that psychiatric measures for depression may not be the most accurate assessment to use for African Americans [8,9,10,11]. Community-based studies frequently refer to other symptoms—not captured in psychiatric measures—that may reflect experiences of psychological distress more accurately, rather than depression, for some African Americans [12]. Though there has not been a comprehensive explanation for the cause and definition of psychological distress, researchers have made various attempts at understanding the term. For example, Decker [13] and Burnette and Mui [14] characterized psychological distress as lack of enthusiasm, problems with sleep (trouble falling asleep or staying asleep), feeling downhearted or blue, feeling hopeless about the future, and feeling emotional (e.g., crying easily or feeling like crying) [12]. 

The paradox is that while Whites tend to report more depressive symptoms on clinical measures of depression, African Americans tend to report more psychological distress on community measures of psychological distress [12,15,16,17]. There is a robust literature on the disparities that exist across race, age, and gender groups for MDD and other psychiatric disorders; however, few studies have examined these disparities for psychological distress and have been able to attest to how psychological distress interferes with the lives of members from various race, age, and gender groups. Using a nationally representative sample, the purpose of this study is to examine the age and gender differences in psychological distress among African Americans and White Americans and consider how much psychological distress interferes with their lives.

Compared to the rest of the nation, some marginalized groups (e.g., racial/ethnic minorities, low socio-economic groups, etc.) have poorer health and are exposed to a broad range of social and environmental factors (e.g., impoverished communities, poor healthcare, etc.) that adversely affect their health [12,15,18,19]. Decades of research have chronicled not only the race and gender differences in physical health but also the mental health outcomes for marginalized racial groups compared to Whites [20,21,22,23]. Despite researchers’ and practitioners’ attention to understanding the biological and psychological circumstances surrounding depression, relatively less emphasis has been placed on the psychosocial characteristics shaped by culture and gender and how this information can inform other measures of poor mental health such as psychological distress. For example, the DSM defines mental disorders such as MDD, but it provides few details about the cultural and gender formulation (i.e., and adherence to gender roles or specific cultural norms) of diagnostic criteria that could potentially modify standard diagnostic practices. Further, since African Americans are less likely to seek mental health care compared to Whites, the experience of psychiatric disorders for African Americans may be less frequently documented in clinical settings. Instead, they may be defined more explicitly by the layperson and occur in community settings; rather than identify this as depression, it is more likely to be identified as stress or psychological distress. 

There are notable differences in psychological distress across various race, gender, and age groups. For example, African Americans experience disproportionately higher levels of psychological distress due to their exposure to a greater frequency and severity of psychosocial stressors (e.g., experiences with marital problems, the justice system, abuse, and financial crises) compared to other groups [15,18,19,24]. Likewise, the frequency and severity of psychosocial stressors are exacerbated by other socio-demographic factors (i.e., age, household income, marital status, education level) that can influence the emotional and psychological health of African Americans. For example, the largest epidemiologic study of African Americans in the United States found that African American men aged 34 and younger experienced psychological distress at higher rates than those over the age of 35 [9,25,26]. 

The current study is guided by the social stress paradigm [27,28,29], which suggests that disadvantaged groups will have poorer mental and physical health than more advantaged groups due to their exposure to more persistent stressors and access to fewer resources [12,28,29]. Similarly, we are also considering the experiences of depression and distress for African Americans and the mental health paradox [12,30,31,32,33], which has been reported in previous studies. Specifically, the mental health paradox acknowledges that African Americans have lower rates of depression compared to Whites, despite high rates of morbidity and mortality associated with preventable health risks. There is a need to understand the effects of psychological distress as well as psychological distress interference as a raced and gendered phenomenon among samples of African Americans and Whites. 

## 2. Materials and Methods

### 2.1. Study Population

The current study utilized a sub-sample from the 2016 National Health Interview Survey (NHIS), an annual survey conducted by the National Center for Health Statistics of the Centers for Disease Control and Prevention with a representative sample of the United States population. Data were collected through in-person interviews [34]. The present study includes a sub-sample of self-identified White (*n* = 26,836, 88%) and African American (*n* = 3777, 12%) adults, between the ages of 18 and 85 (M = 51.67, SD = 18.9). A little more than half the sample (*n* = 16,750, 55%) were women. Due to the use of secondary, de-identified data for this study, IRB review was not required. 

### 2.2. Measures

#### 2.2.1. Psychological Distress 

Psychological distress was measured using the six item Kessler (K-6) Nonspecific Psychological Distress Scale [2,35]. An example item from this scale is, “During the past 30 days, about how often did you feel hopeless?” Participants responded to items using a 5-point Likert scale ranging from 0 (None of the time) to 4 (All of the time). Higher computed scores indicated higher levels of distress. Following the previous use of this measure [2,36], we categorized psychological distress into three groups: low distress level (total scores ranging from 0 to 4), moderate distress level (total scores ranging from 5 to 12), and high distress level (total scores of 13 or higher).

#### 2.2.2. Distress Interference 

The interference of psychological distress on participants’ lives was measured using the follow-up questions to the K-6, “How much did these feelings interfere with your life or activities (during the past thirty days)?” [37] Participants responded using a 4-point Likert scale ranging from 0 (not at all) to 3 (a lot). Higher scores indicated more interference due to reported psychological distress.

### 2.3. Analytic Approach

A one-way analysis of variance (ANOVA) was conducted to evaluate differences in psychological distress between race and age groups. Next, a factorial ANOVA was conducted to compare the main effects of psychological distress, age, and race and the interaction effect between psychological distress, age, and race on psychological distress interference. Psychological distress included three categories (low, moderate, and high); age included three categories (young, middle-aged, and older); and race included two categories (African American and White). Men and women were evaluated independently; thus, only within-gender group comparisons were made. Age groups were determined based on previous psychological distress studies with race and gender groups [9,26]. We used the NHIS computed sample weights for analyses.

## 3. Results

The means and standard deviations for psychological distress are displayed in Table 1. The means and standard deviations for distress interference are displayed in Table 2 for men and women across race, age, and levels of psychological distress. Demographic findings by race, age, and gender have been reported elsewhere [38] and are therefore excluded from this study. 

### 3.1. Psychological Distress among Men and Women

A one-way ANOVA found significant effects of race and age on psychological distress for men and women (Table 3). A post hoc test using the Least Significant Difference (LSD) test indicated varying difference between age and race groups. For example, middle-aged White men (M = 2.55, SD = 3.98) reported significantly more psychological distress than all other White and African American men (Table 1). In contrast, older White men (M = 2.02, SD = 3.52) reported less psychological distress than all other age, race, and gender groups. Among the African American men, older men reported more psychological distress than young men (M = 2.12, SD = 3.4), and middle-aged men (M = 2.16, SD = 3.59). Young White women (M = 3.36, SD = 4.14) reported more psychological distress than all other age, race, and gender groups (Table 1). In contrast, older African American women (M = 2.35, SD = 3.76) reported lower psychological distress than all other White and African American women.

### 3.2. Effects of Psychological Distress on Psychological Distress Interference

Simple effect analyses, using the LSD adjustment, revealed significant mean difference between race and age groups within each level of psychological distress on its interference (Table 1). At low levels of distress, young White men (M = 0.511, SD = 0.73) reported more distress interference than all other men, and young African American women (M = 0.62, SD = 0.84) reported more distress interference than all other women. At moderate levels of distress, older White men (M = 1.21, SD = 0.92) reported higher levels of interference than all other men and young African American women reported higher levels of inference (M = 1.27, SD = 1.01) compared to all the other women. At high levels of distress, young African American men (M = 2.39, SD = 0.74) shared the highest level of interference among all men with middle-aged African American men (M = 2.39, SD = 0.95) and older White men (M = 2.39, SD = 0.84), respectively. Among the women with low and moderate distress, young African American women (M = 0.62, SD = 0.84; M = 1.27, SD = 1.01, low and moderate respectively) reported more interference than all other women and older White women (M = 0.48, SD = 0.74; M = 1.14, SD = 0.92, low and moderate, respectively) reported less interference than all other women. Among the women who reported high levels of distress, middle-age African American women (M = 2.51, SD = 0.78) reported more interference than all other women and young African American women (M = 1.93, SD = 0.95) reported less interference than all women.

A 3 × 3 × 2 factorial ANOVA examined the relationship between distress level, age, and race on psychological distress interference for men (Table 3). There was a main effect of psychological distress, age, and race (Table 3). There was a significant interaction between distress level, gender, and race on distress interference. Essentially, level of psychological distress impacted the level of psychological distress interference differently between age groups and race groups for men. A separate 3 × 3 × 2 factorial ANOVA was conducted to examine the relationship between distress level, age, and race on psychological distress interference for women (Table 3). There was a main effect of psychological distress, age, and race. There was a significant interaction between distress level, gender, and race on distress interference. For women, higher psychological distress was related to more psychological distress interference.

**Figure 1:**
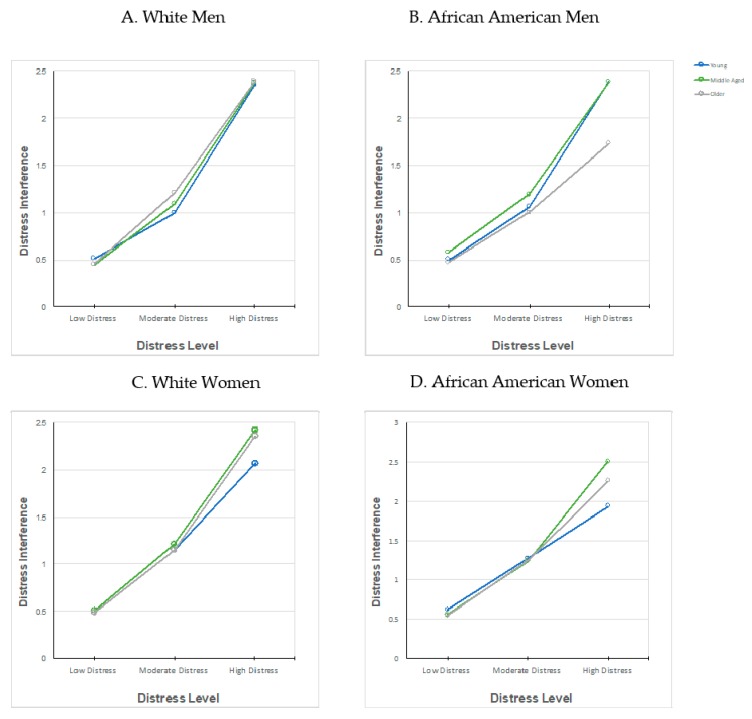
Interaction between distress level and age on distress inference by gender.

In our analysis of the interaction between distress level and age on distress inference by gender, we found that the largest mean difference was among African American men, evident at high levels of distress (see Figure 1B), such that older African American men (M = 1.73, SD = 1.05) reported lower distress than middle-aged African American men (M = 2.39, SD = 0.95), young African American men (M = 2.39, SD = 0.82), and older White men (M = 2.39, SD = 0.84). Among all men, we found those who reported higher levels of distress reported more distress interference with their daily tasks (F (2, 31,932,266) = 2,404,053.36, *p* < 0.001).

## 4. Discussion

The purpose of this paper was to examine age and gender differences in psychological distress among (and how much psychological distress interfered with the lives of) African Americans and Whites. Across all groups, the mean psychological distress scores were low, so we focus our discussion on the within group differences. Our first major finding was that young White women and middle-aged White men experienced higher rates of psychological distress compared to all other race, age, and gender groups. This finding is contrary to the long-standing, consistent findings on psychological distress, which have reported higher rates of psychological distress among African Americans when compared to Whites [7,8,11,25,39]. Despite the plethora of studies supporting this notion, other studies have found that under certain conditions, some Whites experience poorer mental health than Black Americans. For example, Assari and Lankarani [40] found that stress might have a stronger impact on the lives of White men compared to Black men. These mixed findings on psychological distress outcomes call for more research that examines psychological distress beyond race, and instead include outcomes that cut across race, gender, age, and other demographic characteristics. Previous studies have hypothesized why some African Americans who are distressed do not meet diagnostic criteria for depression. For example, some studies have reported that African Americans tend to maintain high levels of hope even in the presence of depressive symptoms and distress, which is different compared to Whites [41]. Similarly, African Americans who are depressed also tend to maintain high levels of positive evaluation toward self, which is protective against distress and depressive symptoms. This is also different for African Americans when compared to Whites [42]. 

Our second major finding was that psychological distress interfered in the lives of all the demographic groups we examined, except for older African American men and young African American women. While the higher reports of interference are not surprising, what is noteworthy is how psychological distress did not influence the lives of older African American men and young African American women in the same way it influenced the lives of the other demographic groups. One interpretation of this finding is that once African American men are in the later stages of life, they may be more accepting of their life transitions and trajectories. This is obviously not the case for all African American men, but there is a literature suggesting that older African American men experience different mental health challenges (e.g., those associated with retirement, functional limitations, loss of friends and loved ones) than their younger counterparts [43], and require coping mechanisms that differ from the ones used in their youth [44]. Similarly, older African American men may be more involved in church and use it as a means of social support compared to younger African American men [45]. As for young African American women, perhaps their resilience to stress at an early age due to supportive adults in the home, school, and community [46] is a protective factor that may waiver as they age. However, this finding would need to be examined more closely for further interpretation. 

The current social, political, and economic climate has underscored the psychosocial stressors faced by marginalized groups, and their mental health outcomes due to these barriers. Studies that consider intersectional implications of not just psychological distress, but the ways it can impact different demographic groups in different ways will be important to future mental health research and practice with marginalized groups. Overall, psychosocial coping and socio-economic challenges faced by marginalized groups have a negative impact on their mental health [9,47,48], and may manifest as psychological distress which, like depression, frequently goes undiagnosed and untreated [49]. 

Finally, our findings suggest that distress may be qualitatively different than depression [12], and under certain conditions, African Americans and Whites may have reverse patterns of MDD and distress that are influenced by demographic characteristics and social determinants of mental health. This supports findings from earlier studies suggesting that depressive symptoms and psychological distress predict risk of MDD for Whites and not African Americans [50]. Findings from the current study are contrary to both the social stress paradigm [12,28,29] and the mental health paradox [12,17,34], because the more historically advantaged groups (Whites) had poorer psychological distress than the more historically disadvantaged groups (African Americans) we tested. Furthermore, we found more differences by gender in psychological distress than by race. 

## 5. Limitations

Our study findings should be interpreted considering a few limitations. For example, our use of the social stress paradigm as a guiding framework may be limited in its application to how we define resources across different demographic groups. For example, while the social stress paradigm focuses on tangible resources, members from some demographic groups may draw from other types of resources, specifically social resources like social support and engagement (e.g., church, importance of family), which we did not measure in this study. A deeper consideration of the various levels and types of resources could better contextualize our results. Second, our decision to categorize the levels of distress could influence how our findings may be interpreted across race, age, and gender. Thirdly, the cross-sectional nature of the National Health Interview Survey does limit our ability to draw causal inferences from the analyses. Finally, the African American sample is representative but the White sample is not. Therefore, the results from the African American sample can be generalizable, but not the results from the White sample. Beyond these limitations, our approach allowed for an exploration of differences in psychological distress and psychological distress interference among various race, age, and gender groups. Our work demonstrates the need for further exploration and future studies on psychological distress that not only considers psychological distress as an outcome, but also the ways psychological distress influences functioning and flourishing among various demographic groups. 

## 6. Conclusions

This study sought to examine psychological distress among a national sample of African Americans and Whites and the ways that psychological distress interfered with their lives. This study was influenced by previous clinical- and community-level studies on depression and psychological distress, as it sought to understand the prevalence and interference of psychological distress among African Americans and Whites. More research is needed to assess specific race, age, and gender disparities across depression and psychological distress using more data sources, and further analyses are needed to assess the influence of these mental health challenges on the daily lives of the people they impact. 

## Figures and Tables

**Table 1:** Means (M) and Standard Deviations (SD) of Psychological Distress.

Age and Race Group	Men		Women	
	M	SD	M	SD
Young White	2.44	3.42	3.36	4.14
Middle-Aged White	2.55	3.97	3.10	4.19
Older White	2.02	3.52	2.66	3.95
Young African American	2.12	3.40	2.84	3.96
Middle-Aged African American	2.16	3.59	3.05	4.45
Older African American	2.23	3.87	2.35	3.76
Total	2.31	3.69	2.95	4.10

**Table 2:** Means and Standard Deviations of Psychological Distress Interference.

Distress Level	Age Group	Race	Men		Women	
			M	SD	M	SD
Low Distress	Young 18 to 30	White	0.51	0.73	0.49	0.65
		African American	0.50	0.72	0.62	0.84
	Middle-Aged 31 to 54	White	0.45	0.74	0.51	0.73
		African American	0.58	0.78	0.56	0.82
	Older 55+	White	0.45	0.72	0.48	0.74
		African American	0.48	0.77	0.54	0.73
Moderate Distress	Young 18 to 30	White	1.00	0.90	1.15	0.89
		African American	1.06	0.97	1.27	1.01
	Middle-Aged 31 to 54	White	1.09	0.92	1.21	0.91
		African American	1.19	0.85	1.23	0.94
	Older 55+	White	1.21	0.92	1.14	0.92
		African American	1.00	0.93	1.25	0.90
High Distress	Young 18 to 30	White	2.36	0.82	2.07	0.91
		African American	2.39	0.74	1.93	0.95
	Middle-Aged 31 to 54	White	2.38	0.93	2.42	0.80
		African American	2.39	0.95	2.51	0.78
	Older 55+	White	2.39	0.84	2.36	0.84
		African American	1.73	1.05	2.25	1.02

Note: At high levels of distress, young and middle-aged African American men do not differ on interference scores. All other cell means are significantly different from each other, by gender.

**Table 3:** Results of ANOVA for Psychological Distress Interference Scores, by Distress, Race, and Age.

**Men ^a^**					
**Source**	**SS**	***df***	**MS**	**F**	**Eta Squared**
Distress	3,377,509.36	2	1,688,754.68	2,404,053.363	0.131
Age	29,066.81	2	14,533.407	20,689.26	0.001
Race	6814.66	1	6814.664	9701.12	0
Distress × Age	33,990.51	4	8497.627	12,096.93	0.002
Distress × Race	16,515.2	2	8257.599	11,755.24	0.001
Age × Race	54,799.86	2	27,399.93	39,005.6	0.002
Distress × Age × Race	26,929.89	4	6732.47	9584.12	0.001
Error	22,431,184.1	31,932,266	0.702		
Total	60,876,378	31,932,284			
**Women ^b^**					
**Source**	**SS**	***df***	**MS**	**F**	**Eta Squared**
Distress	5,193,282.70	2	2,596,641.35	3,713,174.01	0.151
Age	54,433.97	2	27,216.99	38,920.05	0.002
Race	4233.26	1	4233.26	6053.52	0
Distress × Age	82,754.61	4	20,688.65	29,584.59	0.003
Distress × Race	8380.95	2	4190.47	5992.34	0
Age × Race	527.57	2	263.79	377.21	0
Distress × Age × Race	11,335.06	4	2833.76	4052.26	0
Error	29,271,196.26	41,857,550	0.7		
Total	88,253,325	41,857,568			

Note: All F values are significant at *p* < 0.001; ^a^ R Squared = 0.3; ^b^ R squared = 0.31. Abbreviations: SS = Sums of Squares; *df* = Degrees of Freedom; MS = Mean Square.
